# Utilization and quality of primary and specialized palliative homecare in nursing home residents vs. community dwellers: a claims data analysis

**DOI:** 10.1186/s12904-024-01631-z

**Published:** 2025-01-07

**Authors:** Juliane Poeck, Franziska Meissner, Bianka Ditscheid, Markus Krause, Ulrich Wedding, Cordula Gebel, Ursula Marschall, Gabriele Meyer, Werner Schneider, Antje Freytag

**Affiliations:** 1 Institute of General Practice and Family Medicine, Jena University Hospital, Friedrich-Schiller University Jena, Jena, Germany; 2 Department of Palliative Care, Jena University Hospital, Friedrich-Schiller University Jena, Bachstraße 18, Jena, 07743 Germany; 3 BARMER, Wuppertal, Germany; 4 Institute of Health and Nursing Sciences, Martin Luther University Halle-Wittenberg, Halle (Saale), Germany; 5 Center for Interdisciplinary Health Research, University of Augsburg, Augsburg, Germany; 6 Institute for General Practice and Palliative Care, Hannover Medical School, Hannover, Germany

**Keywords:** Palliative care, Primary palliative care, Specialized palliative homecare, Nursing home, End of life, Quality of healthcare, Claims data

## Abstract

**Background:**

There are hardly any data on the extent to which nursing home residents are provided with palliative homecare. We want to add evidence by comparing nursing home residents (who had been living in a nursing home for at least one year) and nursing-care-dependent community dwellers in terms of utilization and quality of palliative homecare.

**Methods:**

We conducted a population-based study with nationwide claims data from deceased beneficiaries of a large German health insurance provider. First, we compared utilization rates of primary palliative care [PPC], specialized palliative homecare [SPHC], and no palliative care [noPC] between nursing home residents and community dwellers, both descriptively and adjusted for covariates. Second, we analyzed the (adjusted) relationship between PPC-only and SPHC (both: starting ≥ 30 days before death), and noPC with healthcare indicators (death in hospital, hospitalization, emergencies, intensive care treatment within the last 30 days of life), and compared these relationships between nursing home residents and community dwellers. Analyses were conducted using simple and multiple logistic regression. Data were standardized by age and gender.

**Results:**

From 117,436 decedents in 2019, 71,803‬ could be included in the first, 55,367‬ in the second analysis. The rate of decedents with noPC was higher in nursing home residents (61.3%) compared to community dwellers (56.6%). Nursing home residents received less SPHC (10.7% vs. 23.2%) but more PPC (30.3% vs. 27.0%) than community dwellers, and achieved better outcomes across all end-of-life healthcare indicators. Adjusted for covariates, both types of palliative homecare were associated with beneficial outcomes, in nursing home residents as well as in community dwellers, with generally better outcomes for SPHC than PPC-only. For most outcomes, the associations with palliative homecare were equal or smaller in nursing home residents than in community dwellers.

**Conclusions:**

The overall better performance in quality of end-of-life care in nursing home residents than in community dwellers may be due to the institutionally provided nursing and general practitioner care within nursing homes. This may also explain higher rates of PPC and lower rates of SPHC in nursing home residents, and why the relationship with both PPC and SPHC are smaller in nursing home residents.

**Trial registration:**

German Clinical Trials Register (DRKS): [DRKS00024133, Date of registration: 28.06.2021].

**Supplementary Information:**

The online version contains supplementary material available at 10.1186/s12904-024-01631-z.

## Background

To some extent, end-of-life care of nursing home residents is considered to be inadequate [1–3]. An important approach to increase quality of end-of-life care in nursing home residents, particularly by avoiding burdensome treatment, is seen in the appropriate involvement of palliative care [1, 4]. International studies indicate that utilization of palliative care in nursing homes has increased [5], but is less common than hospice care [6]. However, little is known about the type of palliative care which is applied in nursing homes [7]. Palliative homecare^1^ includes primary palliative care (PPC) delivered by general practitioners (GPs) mainly supported by outpatient nursing services and specialized palliative homecare (SPHC) provided by specialized teams with (at least) palliative care physicians and specialized nurses with palliative care expertise.

In order to apply adequate measures to enhance quality of end-of-life care in nursing homes, more needs to be known about the actual utilization of palliative care in nursing home residents, and some evidence on the quality of the applied types of palliative care is needed, ideally in comparison to community dwellers.

International studies suggest that palliative care consultations tend to have a positive effect on end-of-life care in nursing home residents, for example in reducing emergency department visits, burdensome care transition and potentially aggressive interventions at the end of life [4, 8, 9]. However, there is no published comparison between different types of palliative homecare, as particularly between PPC and SPHC. To the best of our knowledge, no study has compared the associations of palliative care utilization between nursing home residents and community dwellers, respectively. The two groups differ in functional and cognitive status, for example prevalence of dementia [10], physical fitness level [11], treatment (polypharmacy) and social capital. So far, German studies on end-of-life care in nursing home residents focused on the analysis of hospitalizations and the influence of age and gender [12–14]. In order to fill this gap, we compared nursing home residents vs. community dwellers in two sub-studies addressing the following research questions:

1. How does the rate of utilization of palliative homecare (no PC, PPC and SPHC) differ between nursing home residents and nursing-care-dependent community dwellers?
2. What is the relationship between no PC, PPC-only or SPHC with the quality of end-of-life care (e.g., deaths in hospital, hospitalization, emergencies, intensive care treatment), and how does it differ between nursing home residents and nursing-care-dependent community dwellers?

Our focus is to identify if the setting (nursing home) has a relationship with utilization rate and quality of palliative homecare. For this reason, we consider residents who have lived in the nursing home for a longer period of time.

## Methods

We conducted a population-based study with nationwide claims data from beneficiaries of a major German health insurance provider (BARMER), which covers 10 percent of all persons with statutory health insurance in Germany. The dataset is part of the *pallCompare* project [German Clinical Trials Register (DRKS): DRKS00024133]. The setting includes a total number of 117,436 people aged 19 years or older, who died in 2019, were insured for at least one year before death and resided in Germany.

The claims data set contained pseudonymized demographic information, as well as information on home care and inpatient care, outpatient consultations, hospitalizations, emergency medical services and intensive care treatment.

We standardized all results by age and gender on the level of federal states since BARMER decedents are not equally distributed across all German federal states and to account for known differences in the distribution of key characteristics between BARMER insurants and the general population in Germany [15]. This is a common approach to ensure an adequate level of representativity in studies relying on data from a single insurance provider. We planned, prepared and performed the study and all analyses according to GPS, Good Practice for Secondary data analysis [16]. All statistical analyses were conducted with R (Version 4.1.2). Recommendations of STROSA (consensus German reporting standard for secondary data analyses) [17] and RECORD (reporting of studies conducted using observational routinely collected health data) [18] were followed.

### Methods of research question 1: utilization of palliative homecare in nursing home residents vs. community dwellers

In order to address the first research question, we used claims data to compare the utilization of different forms of palliative homecare between nursing home residents and community dwellers.

**Fig. 1:**
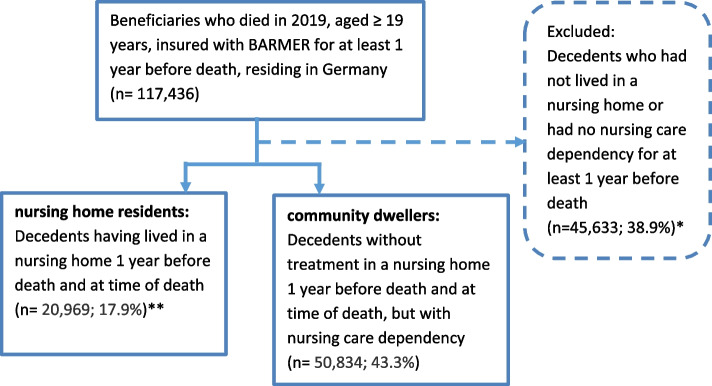
Flow-chart study population research question 1. *Numbers presented in this figure reflect standardized data. Due to rounding, they may not add up precisely to the totals provided. ** Percentages refer to the respective (group) totals provided in previous layers of the chart

We specified our study population for Analysis 1 by including nursing home residents and community dwellers requiring at least a level of care dependency at stage 1.^2^ For the assignment to the groups nursing home residents and community dwellers, the “place of residence” at the time of death and one year before death was determined. This ensures that the setting would have some time to take effect and rules out that results are distorted by residents who entered the nursing home shortly before death. But it does not guarantee continuous nursing home or community residence, just similar residence at two-time points since Individuals with multiple transitions are not identified. The selection of the study population is shown in Fig. 1.

Following the study of Ditscheid et al. (2023) [15], utilization of palliative care was treated in a binary manner (i.e., whether or not patients utilized at least one instance of respective care). More precisely, two different types of palliative homecare (PPC, SPHC) were identified based on documentation of at least one corresponding service for billing purposes in the last year of life. Similar fee schedule items have already been used in other claims data studies to identify services [19, 20]. Further information are provided in Supplementary 1. NoPC was assigned when insurants did not receive any kind of palliative care in the last year of life (neither outpatient nor inpatient [hospital palliative care or hospice care; for identification, see also Ditscheid et al. (2023) [15]).

For our statistical analyses for research question 1, we used simple as well as multiple logistic regression to test for differences in the utilization of two types of palliative homecare (PPC, SPHC) and noPC. In a first step, we used simple logistic regression (predictor: group, i.e., nursing home residents/community dwellers). In a second step, we performed multiple logistic regression including age, gender, cancer diagnosis, comorbidity measured by the Charlson Comorbidity Index [CCI] [21], and urbanity of geographic location in the model as covariates, because previous studies demonstrated correlations between these variables and utilization rates [15, 22]. Although we focused on pure group differences (first step), we provide interested readers with the full picture by adding group differences controlled for covariates in a second step. We report odds ratios (OR) and *p* values for the differences between nursing home residents and community dwellers. Additionally, we report descriptive utilization rates per group (and, for the second step, marginal predictive means as “adjusted rates”). The statistical significance level was set to 5%.

### Methods of research question 2: relationship between palliative homecare and healthcare indicators at the end of life

In Analysis 2, we focused on the relationship between the type of palliative homecare (noPC, PPC-only and SPHC) with healthcare indicators, and explored whether nursing home residents and community dwellers differed with regard to these relationships.

We selected the following healthcare indicators (referring to the last 30 days of life): death in hospital as an indicator of potential underuse of palliative homecare until the end-of-life as an indicator of palliative homecare until the end-of-life, hospitalization, emergency medical services, and intensive care treatment (Table 1). These indicators have been used in other studies to measure quality of end-of-life care [20, 22–28].

**Table 1:** Definition of selected healthcare indicators at the end of life

Healthcare indicator	In claims data identified by
Place of death: hospital	Type of discharge “death” in hospital case data, including death in a palliative care unit (other places of death were considered as “not in a hospital” and include all deaths in a domestic environment: at home, in a nursing home, hospice, or other “domestic” places)
Hospitalization	Begin of a hospitalization within the last 30 days of life, excluding hospital cases with inpatient palliative care
Emergency medical services	Number of days with emergency medical services in the last 30 days of life
Intensive care treatment	Inpatient intensive care treatment during the last 30 days of life

**Fig. 2:**
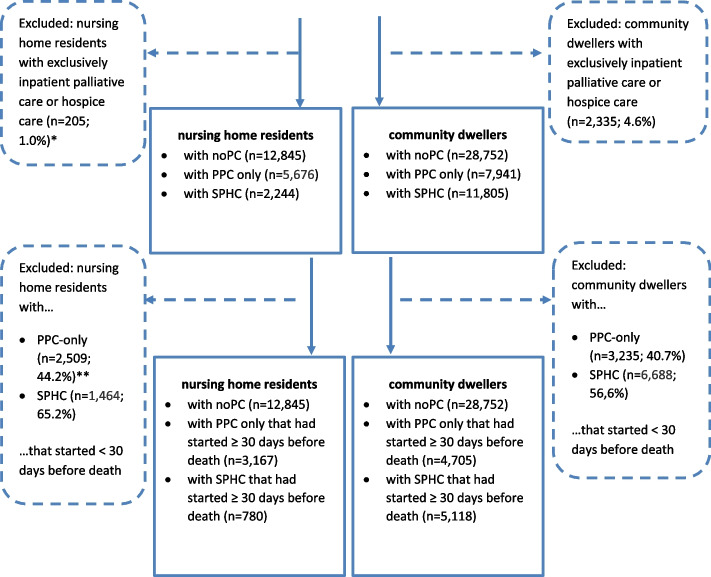
Flow-chart study population research question 2. *Numbers presented in this figure reflect standardized data. Due to rounding, they may not add up precisely to the totals provided. ** Percentages refer to the respective (group) totals provided in previous layers of the chart

In contrast to Analysis 1, we considered distinct groups in Analysis 2. More precisely, we assigned decedents who had received SPHC alone as well as those with both PPC and SPHC to the ‘SPHC’ group, while we assigned decedents who had received PPC alone to the ‘PPC-only’ group (i.e., giving preference to the more comprehensive form of PC, analogous to Krause et al. 2021 [22]), and compared them to decedents who had received noPC at all. To ensure that palliative care could have an association with indicators presented, palliative care had to be documented at least once before the observation period of 30 days before death. Thus, the study population of Analysis 2 is a subset of that of Analysis 1 including all those decedents who belong to one of the six groups: nursing home residents or community dwellers with noPC or PPC-only or SPHC in the last 30 days before death (Fig. 2).

We report rates of healthcare indicators adjusted for relevant covariates (see Analysis 1) as we know from previous studies that the covariates are correlated with the end-of-life healthcare indicators [15, 22, 29].

We applied multiple logistic regression analyses with *group* and *palliative homecare* as well as their interaction as predictors. Entering interaction terms allowed us to check whether the associations between palliative homecare and healthcare indicators vary between nursing home residents and community dwellers. For the predictor *palliative homecare,* we applied backward difference coding, allowing us to test the contrasts of interest (first contrast: PPC-only vs. noPC; second contrast: SPHC vs. PPC-only). For the predictor *group*, we applied effect coding, allowing us to check the overall relationship between palliative care and healthcare indicators, averaged across both nursing home residents and community dwellers. We report OR and *p* values for all associations, and marginal predictive means as adjusted rates. The statistical significance level was set to 5%.

## Results

### Results of research question 1: utilization of palliative homecare in nursing home residents vs. community dwellers

From 117,436 individuals deceased in 2019, 71,803‬ were included (Fig. 1). On average, nursing home residents were older, more often female, had more seldom a cancer diagnosis and had less comorbidities than community dwellers. As expected, nursing home residents were dependent on higher levels of nursing care than community dwellers (Table 2).

**Table 2:** Study population for research question 1

	**Statistics**	nursing home residents	community dwellers
**Total number of decedents in each group**	N	20,969	50,834
**Age (years)**	Mean (SD)	86.3 (± 9.3)	79.5 (± 11.5)
** Age group < 65 years**	n (%)	708 (3.4%)	5,711 (11.2%)
** Age group 65–74 years**	n (%)	1,114 (5.3%)	7,791 (15.3%)
** Age group 75–84 years**	n (%)	5,448 (26.0%)	18,314 (36.0%)
** Age group 85–94 years**	n (%)	10,374 (49.5%)	16,474 (32.4%)
** Age group 95 + years**	n (%)	3,324 (15.9%)	2,545 (5.0%)
**Female**	n (%)	14,988 (71.5%)	24,415 (48.0%)
**Cancer diagnosis**	n (%)	4,686 (22.3%)	25,842 (50.8%)
**Charlson Comorbidity Index (CCI)**	Mean (SD)	5.2 (± 3.2)	7.5 (± 4.1)
**Highest level of nursing care within the last year of life**	Mean (SD)	4.1 (± 0.9)	3.3 (± 1.1)
**Level of nursing care**			
** 1**	n (%)	21 (0.1%)	1,876 (3.7%)
** 2**	n (%)	1,408 (6.7%)	12,563 (24.7%)
** 3**	n (%)	3,906 (18.6%)	14,230 (28.0%)
** 4**	n (%)	7,612 (36.3%)	13,520 (26.6%)
** 5**	n (%)	8,022 (38.3%)	8,646 (17.0%)
**Residency, urban**	n (%)	14,454 (68.9%)	34,861 (68.6%)

Utilization of palliative care differed significantly between nursing home residents home and community dwellers (Table 3). More precisely, the rate of decedents who received no palliative care was higher in nursing home residents compared to community dwellers (OR = 1.21, *p* < 0.001). Interestingly, when controlling for covariates, the effect was reversed. Nursing home residents obtained more primary palliative care (OR = 1.19, *p* < 0.001) and less specialized palliative homecare (OR = 0.40, *p* < 0.001) than community dwellers, also when adjusting for covariates.

**Table 3:** Utilization of palliative homecare

**Group**				**Statistics**		
	**nursing home residents**	**community dwellers**	**OR**	**95% CI**		**p**
**No palliative care**	61.3%	56.6%	1.21	[1.18, 1.25]		< .001
^ a^*with covariates controlled for:*	*52.5%*	*60.1%*	*0.70*	[0.67, 0.73]		< *.001*
**Primary palliative care (PPC)**	33.4%	29.6%	1.19	[1.15, 1.24]		< .001
^ a^*with covariates controlled for:*	*39.0%*	*27.6%*	*1.74*	[1.68, 1.81]		< *.001*
**Specialized palliative homecare (SPHC)**	10.7%	23.2%	0.40	[0.38, 0.42]		< .001
^ a^*with covariates controlled for:*	*16.9%*	*20.3%*	*0.78*	[0.74, 0.82]		< *.001*

Differences also emerged in the average duration of palliative homecare before death. Nursing home residents receive PPC and SPHC much later on average (Supplementary 2, Table S1)

^a^Multiple logistic regression including covariates age, gender, cancer diagnosis, comorbidity

### Results of research question 2: relationship between palliative homecare and healthcare indicators at the end of life

From 71,803 individuals deceased in 2019 and included in the first analysis, 55,367‬ were included in the second analysis (Fig. 2). More than 50% of those who received palliative homecare within the last year of life, were excluded because palliative care started less than 30 days before death. Similar to the results of research question 1, group characteristics differed descriptively in age, sex, prevalence of cancer diagnosis and comorbidity. In both nursing home residents and community dwellers, cancer diagnosis appeared more often in decedents utilizing SPHC (Table 4).

**Table 4:** Study population for research question 2

	**Statistics**	***nursing home residents***			***community dwellers***		
		***noPC***	***PPC-only***	***SPHC***	***noPC***	***PPC-only***	***SPHC***
**Total number of decedents in each group**	N	12,845	3,167	780	28,752	4,705	5,118
**Age (years)**	Mean (SD)	86.3 (± 9.4)	86.9 (± 9.0)	82.8 (± 11.6)	81.4 (± 10.6)	79.0 (± 11.5)	73.2 (± 12.5)
**Female**	n (%)	9,149 (71.2%)	2,305 (72.8%)	548 (70.3%)	14,126 (49.1%)	2,203 (46.8%)	2,332 (45.6%)
**Cancer diagnosis**	n (%)	2,407 (18.7%)	843 (26.6%)	330 (42.4%)	9,566 (33.3%)	3,169 (67.4%)	4,463 (87.2%)
**Charlson Comorbidity Index (CCI)**	Mean (SD)	4.9 (± 3.0)	5.4 (± 3.4)	6.6 (± 4.0)	6.3 (± 3.7)	8.8 (± 4.2)	9.7 (± 3.7)
**Highest level of nursing care within the last year of life**	Mean (SD)	4.0 (± 0.9)	4.2 (± 0.9)	4.2 (± 0.9)	3.1 (± 1.1)	3.5 (± 1.1)	3.7 (± 1.0)
**Level of nursing care**							
**1**	n (%)	13 (0.1%)	3 (0.1%)	1 (0.1%)	1,548 (5.4%)	115 (2.5%)	43 (0.8%)
**2**	n (%)	974 (7.6%)	142 (4.5%)	33 (4.2%)	8,893 (30.9%)	968 (20.6%)	620 (12.1%)
**3**	n (%)	2,623 (20.4%)	442 (14.0%)	126 (16.1%)	8,395 (29.2%)	1,293 (27.5%)	1,389 (27.1%)
**4**	n (%)	4,733 (36.8%)	1,053 (33.2%)	262 (33.6%)	6,331 (22.0%)	1,335 (28.4%)	1,809 (35.4%)
**5**	n (%)	4,502 (35.1%)	1,527 (48.2%)	359 (46.0%)	3,585 (12.5%)	994 (21.1%)	1,256 (24.5%)
**Residency, urban**	n (%)	8,684 (67.6%)	2,194 (69.3%)	595 (76.3%)	20,007 (69.6%)	3,085 (65.6%)	3,377 (66.0%)

**Fig. 3:**
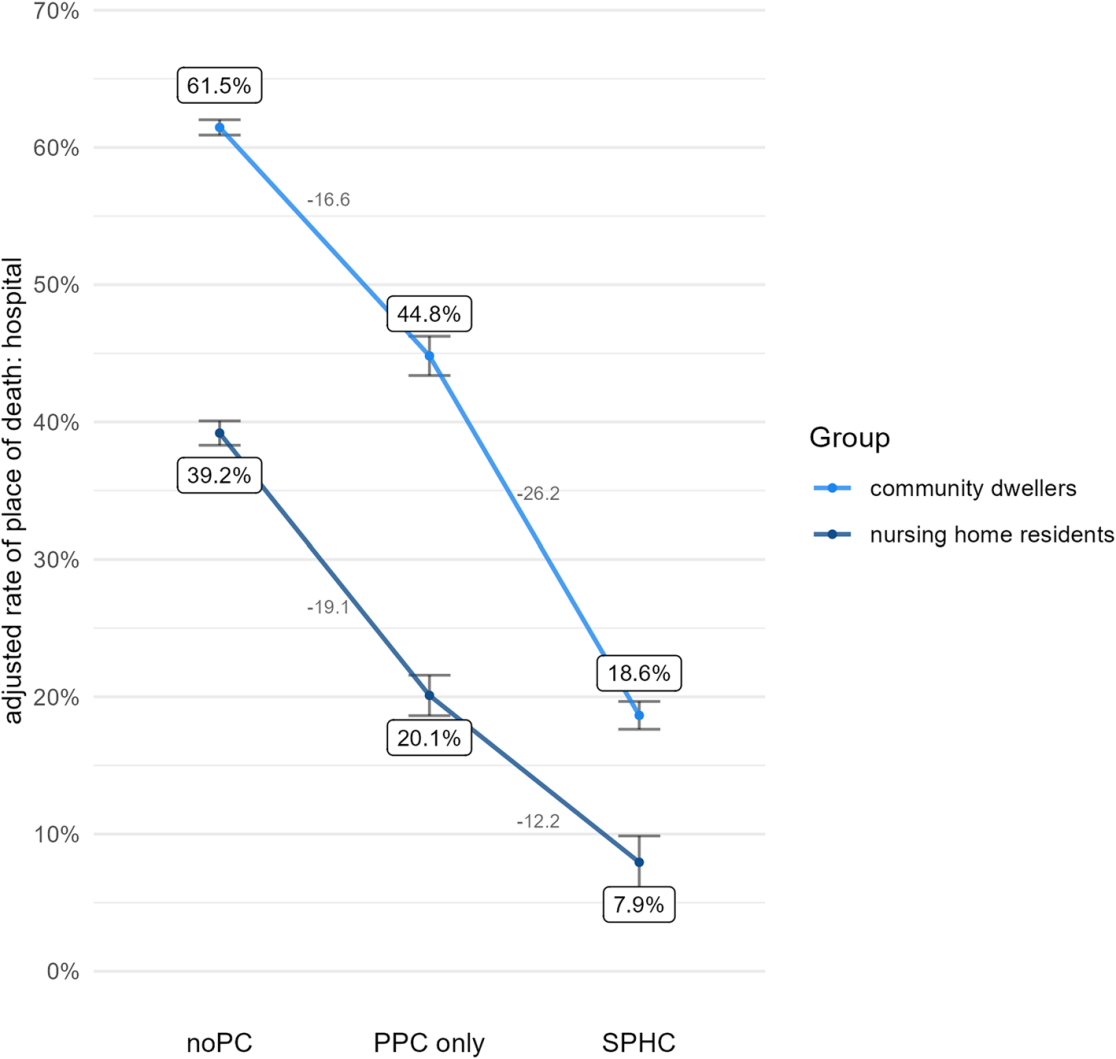
Adjusted rate of place of death: hospital

**Fig. 4:**
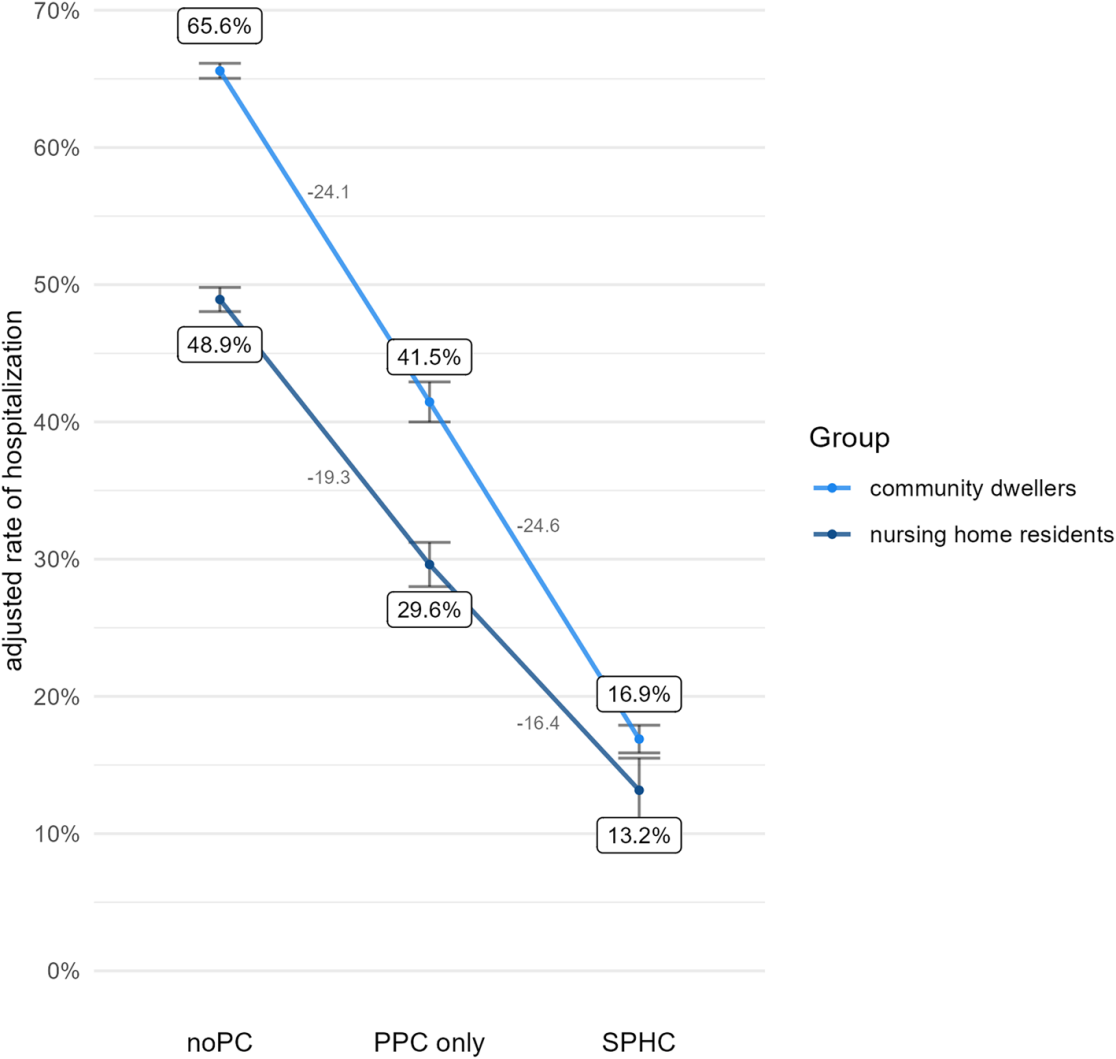
Adjusted rate of hospitalization

**Fig. 5:**
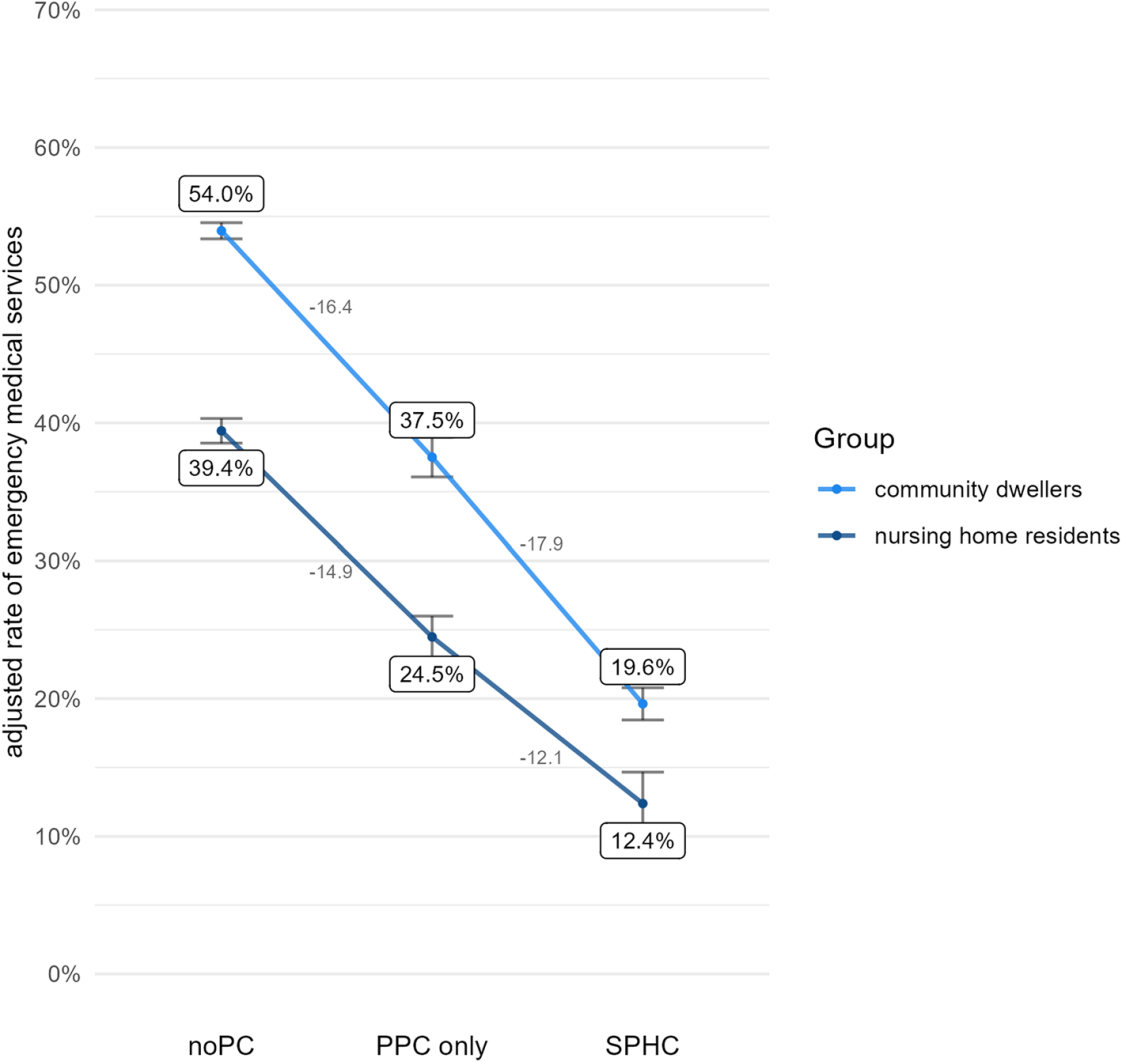
Adjusted rate of emergency medical services

**Fig. 6:**
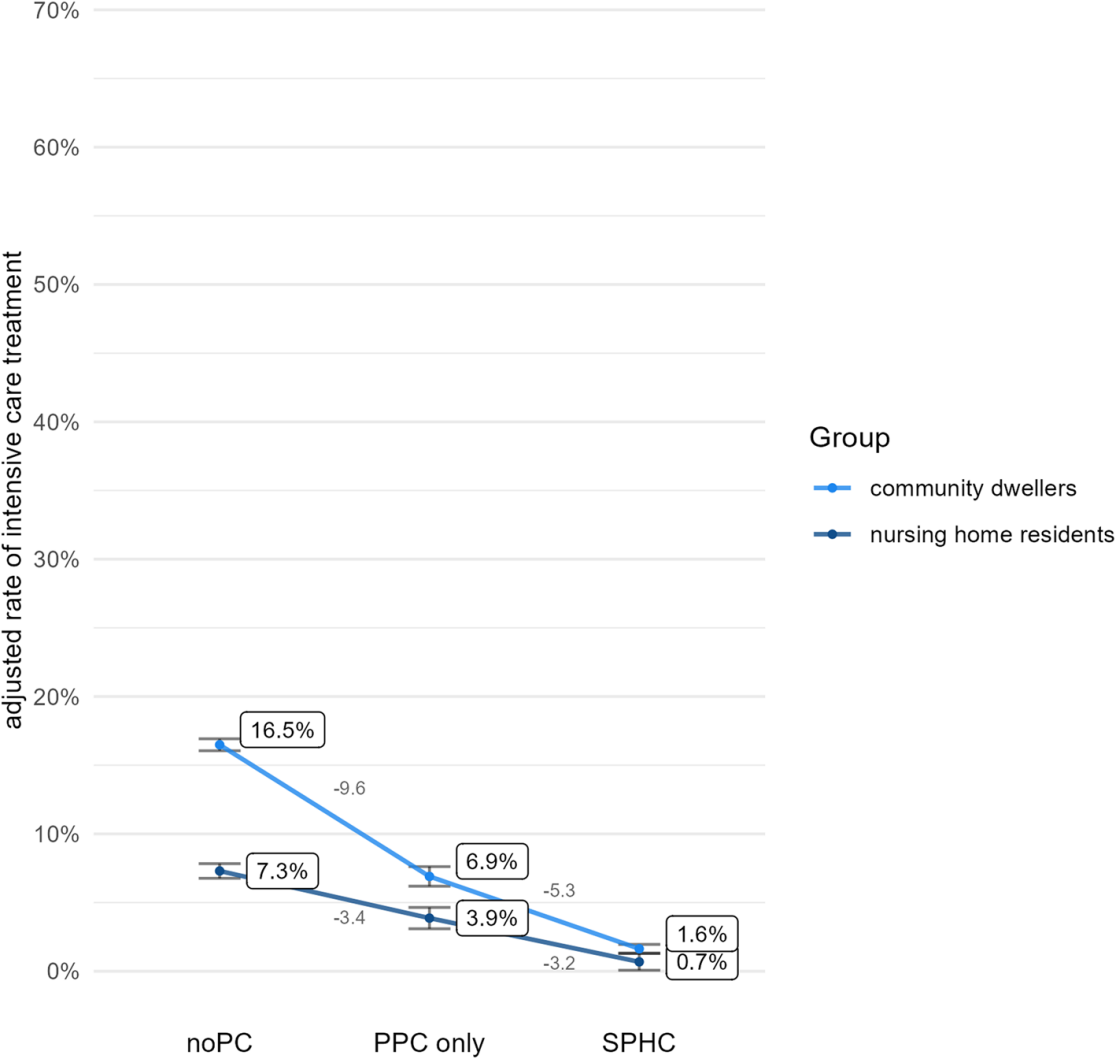
Adjusted rate of intensive care treatment

Figures 3, 4, 5 and 6 illustrate the results for each health care indicator. Altogether, nursing home residents show better outcomes than community dwellers across both types of palliative homecare and also compared with noPC. However, these findings where qualified when examining how strong PPC-only vs. noPC and SPHC vs. PPC-only affect the outcomes in both groups.

### Place of death: hospital

On average, decedents with PPC-only had a lower probability of dying in hospital than decedents with noPC (OR = 0.42, *p* < 0.001). However, as revealed by a significant interaction term (*p* < 0.001), the association differs between nursing home residents and community dwellers. To follow up on this finding, we conducted simple slope analyses and found that PPC-only is more powerful in reducing the rate of in-hospital-deaths among nursing home residents (OR = 0.36, *p* < 0.001) than among community dwellers (OR = 0.48, *p* < 0.001).

On average, decedents having received SPHC had a lower probability of dying in hospital than decedents having received PPC-only (OR 0.29, *p* < 0.001). A non-significant interaction term (*p* = 0.114) indicated that this relationship does not differ between nursing home residents and community dwellers.

### Hospitalization

Decedents having received PPC-only had a lower probability of hospitalization within the last 30 days of life than decedents with noPC (OR = 0.38, *p* < 0.001). The significant interaction term (*p* = 0.002) reveals that PPC-only has reduced the rate of hospitalization to a smaller extent among nursing home residents (OR = 0.42, *p* < 0.001) compared to community dwellers (OR = 0.35, *p* < 0.001).

Decedents having received SPHC had a lower probability of hospitalization than decedents having received PPC-only (OR = 0.30, *p* < 0.001). A group difference between nursing home residents and community dwellers could be found (interaction term, *p* = 0.047). SPHC appeared to be less powerful in reducing hospital treatment (as compared to PPC-only) among nursing home residents (OR = 0.34, *p* < 0.001) vs. community dwellers (OR = 0.27, *p* < 0.001).

### Emergency medical services

The incidence of using emergency medical services was lower for decedents with PPC than with noPC (OR = 0.50, *p* < 0.001). According to a non-significant interaction term (*p* = 0.631), the associations did not differ between nursing home residents and community dwellers.

Decedents within SPHC had a lower risk of obtaining emergency medical services than decedents within PPC-only (OR = 0.42, *p* < 0.001). Due to non-significant interaction term (*p* = 0.565), no group differences were seen between nursing home residents and community dwellers with regard to the relationship of SPHC vs. PPC.

### Intensive care treatment

The risk of getting intensive care treatment was lower for decedents with PPC-only than with noPC (OR = 0.43, *p* < 0.001). Group difference appeared between nursing home residents and community dwellers (interaction term, *p* = 0.011). PPC-only decreased the rate of intensive care treatment (as compared to noPC) less among nursing home residents (OR = 0.51, *p* < 0.001) vs. community dwellers (OR = 0.36, *p* < 0.001).

The risk of receiving intensive care treatment was lower for decedents with SPHC than with PPC-only (OR = 0.19, *p* < 0.001). A non-significant interaction term (*p* = 0.585) shows that this association does not differ between nursing home residents and community dwellers.

## Discussion

This is the first analysis comparing nursing home residents and community dwellers in terms of utilization and quality of palliative homecare. Nursing home residents receive less palliative care in total, less SPHC but more PPC compared to community dwellers. Palliative homecare demonstrates a positive association with all healthcare indicators with SPHC being more beneficial than PPC. The benefit of palliative homecare was evident in nursing home residents and community dwellers. Investigating differences in the relationship between palliative homecare and healthcare indicators, group differences were identified for certain quality indicators. PPC-only vs. noPC can be more powerful in reducing the rates of hospitalization and intensive care treatment in community dwellers. In contrast, it seems to be more beneficial in reducing the rate of in-hospital deaths (including inpatient palliative care) among nursing home residents living 1 year in nursing home. Comparing the relationships of SPHC to PPC-only, the rate of hospitalization decreased to a greater extent in community dwellers. Nevertheless, nursing home residents demonstrate better outcomes than community dwellers, across all types of palliative homecare and with noPC.

### Differences in sociodemographic characteristics

Sociodemographic characteristics varied considerably between nursing home residents and community dwellers. As expected, average level of nursing care was higher in nursing home residents. Moreover, nursing home residents were older and more often female, which has also been reported in other studies [12, 13]. Nursing home residents showed a noticeably lower proportion of cancer diagnoses, possibly due to nursing home residents receiving fewer consultations from medical specialist than community dwellers [30, 31]. Presumably, at an advanced age (> 80 years), less medical diagnostics are conducted. Therefore, some diseases remain undetected. This might also explain why nursing home residents have a lower comorbidity index. These differences in patient characteristics influenced utilization rates of palliative care as well as healthcare indicators (see following sections).

### Differences in the utilization of palliative homecare

Differences in sociodemographic characteristics (particularly in cancer diagnosis and proportion of female sex) have an association with the utilization rate for those without palliative care, explaining why the association is reversed when these characteristics are controlled for. Our study revealed a lower utilization of SPHC in nursing home residents compared to community dwellers. And SPHC as well as PPC is involved later in nursing home residents than in community dwellers. This also applies when adjusted for the occurrence of a cancer diagnosis (cancer rate is lower in nursing home residents). At the same time, utilization of PPC was higher in nursing home residents. One possible reason for the lower utilization rate of SPHC and the later involvement in nursing home residents might be that a certain amount of palliative care is already provided in nursing homes. In Germany, a number of legislative changes have been initiated, e.g., social law supports the implementation of advanced care planning for the last stage of life in nursing homes (§ 132g SGB V) and end-of-life care is explicitly included in nursing care services (SGB XI § 28). Recent studies from Germany point out that the majority of nursing homes report to have developed concepts for hospice culture and palliative care competence and to have established them organizationally [32]. However, case studies revealed large differences in the implementation of such concepts [33] and largely varying numbers of employees with basic or advanced education in palliative care in facilities [34]. Unfortunately, based on our data, it is not possible to reveal the frequency of palliative care provided by nurses in the care facilities. On the other hand, the higher utilization of PPC in nursing home residents could be explained by the fact that many nursing home residents are frequently monitored by GPs who might document and bill PPC when seeing the patient. Together, the utilization of both of these types of palliative care in nursing home residents – the one delivered by nurses inside the nursing home and the one delivered by the attending GP as PPC – might be the reason why SPHC is being applied less often in nursing home residents than in community dwellers.

### Differences in the quality of end-of-life care

Our study provides evidence of a relationship between palliative homecare and quality of end-of-life care. The benefit of PPC-only vs. noPC and SPHC vs. PCC-only was shown in both settings, nursing home residents and community dwellers. This corresponds to the general finding that specialized palliative homecare seems to be more powerful in reducing the rate of potentially aggressive end-of-life interventions than PPC alone [22].

A secondary result of Krause et al. (2021) [22] was that living in a nursing-home was linked to less aggressive therapies at the end of life. This is consistent with our general finding that nursing home residents showed better outcomes than community dwellers whether palliative care has been provided or not. Adequate care is more difficult to ensure in a domestic environment, despite the support provided by palliative services. This observation supports the notion that a certain amount of (internal) palliative care is already being provided in nursing homes and it is consequential to assume a positive relationship with quality of end-of-life care. Nursing homes provide 24-h skilled care for patients, which is not available to the same extent in non-nursing home residents settings. Some studies suggest that identifying palliative care needs in nursing homes depends on nurses' assessment, experience and qualifications [33, 35]. However, it is well known that there is a huge and increasing shortage of qualified nursing staff and that there is a wide variation in the quality of nursing homes. The pandemic has further exacerbated the workforce situation [36, 37]. Workload and time constraints in palliative care are high [38]. This highlights the importance of educating and sensitizing nursing staff.

Another result of our study is that—depending on the setting—the two types of (additional external) palliative homecare affect healthcare indicators in different ways. The most striking differences are that the PPC association with reduced hospital deaths seems to be stronger, whereas that of SPHC weaker in nursing home residents vs. community dwellers. These findings are not directly intuitive and need further exploration. Overall, the association with additional external homecare appears to be rather lower in nursing home residents than in community dwellers. On the one hand, this may be due to the fact that the basic nursing support of community dwellers is lower than that of nursing home residents. On the other hand, the comparatively lower level of aggressive end-of-life care in nursing home residents is more difficult to be lowered further by palliative care.

### Strengths and limitations

An important limitation results from the inclusion and exclusion criteria for the group of nursing home residents. We decided to include nursing home residents, living in a nursing home at the time of death as well as one year before death. This implies that a considerably smaller number of nursing home residents was included (*n* = 20,969) than if nursing home residents at the time of death only had been included (*n* = 36.698). However, we particularly aimed at identifying wether the “place of residence” has an influence on utilization and quality of palliative homecare and the setting might need some time to take effect. Therefore, the applied inclusion criterion allows a clean separation of the groups, as residents were excluded who entered the nursing home shortly before death and/or where palliative care was initiated before entering the nursing home. Nevertheless, results are likely to change depending on inclusion criteria. Our definition does not guarantee a continuous nursing home residence for one year, since individuals with multiple transitions are not identified and not accounted for in our analyses. That is why individuals who resided in a nursing home one year before death and at their death but in between went back and forth between community and nursing home settings are regarded as having lived in a nursing home throughout the year (same applies to community dwellers vice versa). Therefore it is important to point out, that our results apply only to the selected subset of the nursing home residents. The sensitivity analyses with other variables for nursing home residents is presented in the Supplementary 3. A further, but milder limitation of Study 2 was the additional exclusion of nursing home residents who had received palliative homecare for a shorter period than 30 days before death. This criterion is owed to the selected outcome indicators referring to the last 30 days of life correspondingly. That this time restriction (compared to e.g. 14 days) does not have relevant impact on the results, could be shown by our previous study [22].

Beside the well-known limitations of claims data (e.g., no patient-reported outcomes; documentation for billing, not for study purposes), it is important to realize that the data do not allow for identifying the amount and intensity of palliative care that is delivered within nursing homes. The same holds for the intensity of (external) palliative homecare which – in contrast to simple frequency – is not measureable in a valid way. Factors other than those considered might influence outcomes that are not included in our data, such as individual preferences, personal values and particularly the amount of social support an individual patient receives beyond any formal palliative care service, e.g., as provided by family members. Another important and common limitation is, that the data can be compromised by incomplete documentation, particularly related to PPC. For example, PPC services GPs provided without billing them (for different reasons) cannot be captured in the analysis.

Due to the potential confounders we could not observe and adjust for, our approach to identify causal effects of palliative care is limited. Furthermore, it is obvious that we do not give a complete picture of end-of-life care quality by the four healthcare indicators we selected. Future studies should investigate the working mechanism of palliative homecare in two settings (nursing home and domestic care) and deliver more data on its effect regarding burdening trajectories of end-of-life care and quality of life before death. Our results may have limited applicability to other countries due to the specificities of the German healthcare system (e.g. outpatient care, other approaches to palliative care). Nevertheless, the strength of our claims data study (which is equal to a full census study in terms of the respective health insurance fund) lies in the still large number of patients analyzed and the validity of the measured outcome indicators.

## Conclusions

The overall better performance in quality of end-of-life care in nursing home residents than in community dwellers may be due to the institutionally provided nursing and GP care within nursing homes. This may also explain higher rates of PPC billed by GPs and lower rates of SPHC delivered by specialized teams in nursing home residents, and, acknowledging the association of PPC and especially SPHC in both groups, why it is smaller in this subset of nursing home residents than in community dwellers.

This might point into the direction that in a world of scarce resources, SPHC should focus more on community dwellers, and in nursing homes, palliative care should be integrated early into the regular workflows of nursing homes [39, 40]. Necessary requirements are further training for nursing staff in palliative care [1, 34] and reliable medical support of nursing staff.

## Supplementary Information

Supplementary Material 1.

## Data Availability

Access to data is only provided through data use agreement and via protected gateway to the scientific data warehouse of the health insurance fund BARMER. Further information about the data and access regulations are available from the corresponding author on request.

## Abbreviations

PPC: Primary palliative care
SPHC: Specialized palliative homecare
NoPC: No palliative care
GPs: General practitioners

## Footnotes

1 palliative homecare defined as outpatient palliative care (in contrast to hospital- and hospice-palliative care).

2 Care dependency was assessed by the Medical Service of German Statutory Health Insurance providers. Persons are considered with a level of care dependency if they have health-related impairments in their independence or abilities and therefore require nursing assistance. Depending on the severity, a classification is made between care level 1 to 5 (1 = minor, 2 = significant, 3 = severe, 4 = more severe, 5 = most severe impairment of independence or abilities with special requirements for nursing care). With repeated assessments, the level can change across time. For the study, we chose the persons highest documented level of care dependency in the year before death.
