# Supplementary material for: Utilization and quality of primary and specialized palliative homecare in nursing home residents vs. community dwellers: a claims data analysis
Source: BMC Palliat Care. 2025 Jan 7;24:3. doi: 10.1186/s12904-024-01631-z (PMC11706154; doi:10.1186/s12904-024-01631-z)
Supplement: Supplementary file 1 — Supplementary Material 1. [file 12904_2024_1631_MOESM1_ESM.docx]

## **Supplementary Information**

**Utilization and quality of primary and specialized palliative homecare in nursing home residents vs. community dwellers: a claims data analysis**

Juliane Poeck^1^, Franziska Meissner^1^, Bianka Ditscheid^1^, Markus Krause^1^, Ulrich Wedding^2^, Cordula Gebel², Ursula Marschall³, Gabriele Meyer^4^, Werner Schneider^5^, Antje Freytag^1^

1 Institute of General Practice and Family Medicine, Jena **University Hospital, Friedrich-Schiller University Jena, Jena, Germany**

2 Department of Palliative Care, **Jena University Hospital, Friedrich-Schiller University Jena, Jena, Germany**

3 BARMER, Wuppertal, Germany

4 Institute of Health and Nursing Sciences, Martin Luther University Halle-Wittenberg, Halle (Saale), Germany

**5 Center for Interdisciplinary Health Research, University of Augsburg, Augsburg, Germany**

**Corresponding author**

PD Dr. Antje Freytag

**Institute of General Practice and Family Medicine**

**University Hospital Jena, Friedrich Schiller University**

Bachstraße 18

07743 Jena, Germany

Tel.: +49 3641 9395811

E-Mail: [Antje.Freytag@med.uni-jena.de](mailto:Antje.Freytag@med.uni-jena.de)

## **Supplementary 1: Identification of primary palliative care and specialized palliative homecare**

**Primary palliative care** was identified via the fee schedule items (GOP) of the Uniform Value Scale (EBM): 03370/04370, 03371/04371, 03000/04000, 03372/04372, 01410 or 01413, 03373/04373. Only general practitioners (and pediatricians) can bill these items. According to the oncology agreement^[[1]](#footnote-1)^, oncologists in Germany can bill the palliative care code 86518. Additional items by regional Associations of Statutory Health Insurance Physicians (KV) or selective-contracting were extensively researched and used to identify primary palliative care, see Ditscheid et al. (2023), Supplement p. 5-7. We identified the utilization of particularly qualified and coordinated palliative care (BQKPmV) via the nationally valid EBM-GOP 37300, 37302, 37305, 37306, 37314, 37317, 37318 and 37320 (Annex 30 to the BMV-Ä). For our analyses, BQKPmV was assigned to primary palliative care.

**Specialized palliative homecare** was identified via the EBM-GOP: 01425, 01426. In addition, the health insurance funds (BARMER) provided us with a special table containing SPHC services billings. Items by regional Associations of Statutory Health Insurance Physicians (KV) or selective-contracting with billing of services were extensively researched to identify specialized palliative homecare. For detailed information, see Ditscheid et al. (2023), Supplement p. 7-9.

## **Supplementary 2: Onset of initiating palliative care**

Table S1 shows the characteristics of the onset of initiating palliative care in the two groups as we defined them in the main paper (place of residence one year before death AND at the time of death).

*Table S1. Average duration/onset of initiating palliative care in days before death*

|  | Mean (SD) |  | Median (IQR) |
| --- | --- | --- | --- |
| *Primary palliative care (PPC)* |  |  |  |
| nursing home residents | 110 (+/-130) |  | 41 (8,24) |
| community dwellers | 109 (+/-116) |  | 55 (15,19) |
|  |  |  |  |
| *Specialized palliative homecare (SPHC)* | |  |  |
| nursing home residents | 55 (+/-88) |  | 14 (4,61) |
| community dwellers | 58 (+/-81) |  | 24 (7,71) |

*SD – standard derivation; IQR – interquartile relation*

## **Supplementary 3: Sensitivity Analyses with a different definition of the two groups (place of residence at time of death only)**

In the following section we present post-hoc analyses we performed to check whether our results would hold when we assign individuals to the groups “nursing home residents” vs. “community dwellers” according to their place of residence at the time of death only (i.e., disregarding their place of residence one year before death).

**Study Population**: **Utilization of palliative homecare in nursing home residents vs. community dwellers (*place of residence at time of death*)**

Table S2 presents the study population for research question 1 when defining the groups on the place of residence at time of death only. Compared with those who lived in a nursing home for at least the last year of their life (see Table 1 in the main article), people who entered a nursing home during the last year of their life are slightly more likely to be male, more likely to have cancer and have a slightly higher overall comorbidity rate. In general, the two groups tend to be more similar to each other when applying the alternative definition (i.e., taking into account only the place of residence at the time of death) as compared to the rather strict definition presented in the main article.

*Table S2. Study population for research question 1 based on place of residence at time of death only*

|  | Statistics | nursing home residents *(at time of death only)* | community dwellers *(at time of death only)* |
| --- | --- | --- | --- |
| Total number of decedents in each group | N | 36,698 | 51,030 |
| Age (years) | Mean (SD) | 84.7 (9.8) | 79.5 (11.5) |
| Age group <65 years | n (%) | 1,685 (4.6%) | 5,734 (11.2%) |
| Age group 65-74 years | n (%) | 2,667 (7.3%) | 7,818 (15.3%) |
| Age group 75-84 years | n (%) | 10,989 (29.9%) | 18,366 (36.0%) |
| Age group 85-94 years | n (%) | 17,001 (46.3%) | 16,551 (32.4%) |
| Age group 95+ years | n (%) | 4,335 (11.9%) | 2,560 (5.0%) |
| Female | n (%) | 24,167 (65.9%) | 24,529 (48.1%) |
| Cancer diagnosis | n (%) | 12,165 (33.1%) | 25,886 (50.7%) |
| Charlson Comorbidity Index (CCI) | Mean (SD) | 6.1 (+/- 3.7) | 7.5 (+/- 4.1) |
| Highest level of nursing care within the last year of life | Mean (SD) | 3.9 (+/- 1.0) | 3.3 (+/- 1.1) |
| Level of nursing care |  |  |  |
| 1 | n (%) | 58 (0.2%) | 1,876 (3.7%) |
| 2 | n (%) | 3,263 (8.9%) | 12,583 (24.7%) |
| 3 | n (%) | 8,475 (23.1%) | 14,275 (28.0%) |
| 4 | n (%) | 13,429 (36.6%) | 13,584 (26.6%) |
| 5 | n (%) | 11.473 (31.3%) | 8,711 (17.1%) |
| Residency, urban | n (%) | 25,414 (69.3%) | 35,009 (68.6%) |

**Results: Utilization of palliative homecare in nursing home residents vs. community dwellers (*place of residence at time of death*)**

The main conclusions regarding utilization of primary palliative care (PPC) and specialized palliative homecare (SPHC) do not change with the alternative definition of the subgroups: Still, we found that nursing home residents descriptively utilized less SPHC but more PPC than community dwellers (see Table S3). However, while adjusting for age, gender, cancer diagnosis and comorbidity did not change this conclusion in the main analysis (see Table 2 in the main article), this was the case when applying the alternative definition of the subgroups. Here, we found that the adjusted utilization rates of both forms outpatient PC (i.e., PPC and SPHC) were higher for nursing home residents than for community dwellers. Moreover, the descriptive (as well as the adjusted) rate of individuals who did not utilize any form of palliative care in their last year of life was lower for nursing home residents as compared to community dwellers when applying the alternative definition of subgroups. To sum up, while the main conclusions regarding research question 1 still hold, some details changed, and the absolute difference in rates between the two subgroups turned out to be smaller. Given the fact that alternative definition blurs the line between the two subgroups (see above), this was an expected result.

*Table S3. Utilization of palliative homecare (place of residence at time of death)*

| Group | | |  | Statistics | | |
| --- | --- | --- | --- | --- | --- | --- |
|  | **nursing home residents** | **community dwellers** | **OR** | | **95% CI** | **P** |
| No palliative care | 55.1% | 56.6% | 0.94 | | [0.93, 0.97] | < .001 |
| **with covariates controlled for:* | *50.4%* | *59.9%* | *0.64* | | [0.62, 0.66] | *< .001* |
|  |  |  |  | |  |  |
| Primary palliative care (PPC) | *36.9*% | 29.6% | 1.39 | | [1.35, 1.43] | < .001 |
| **with covariates controlled for:* | *39.9%* | *27.8%* | *1.80* | | [1.74, 1.85] | *< .001* |
|  |  |  |  | |  |  |
| Specialized palliative homecare (SPHC) | 17.0% | 23.2% | 0.68 | | [0.66, 0.70] | < .001 |
| **with covariates controlled for:* | *21.3%* | *20.2%* | *1.07* | | [1.03, 1.12] | *< .001* |

**Results: Relationship between palliative homecare and healthcare indicators at the end of life (*place of residence at time of death*)**

Concerning the relationship between quality and outpatient palliative care, the overall picture was the same: when applying the alternative definition of subgroups, main conclusions still hold but the details turn out to be a bit blurry. More precisely, we still found that palliative homecare was associated with beneficial outcomes in both nursing home residents and community dwellers. As compared to individuals with noPC, we observed smaller rates of hospitalization, intensive care treatment, emergency medical services, and in-hospital deaths for individuals with PPC-only, and even smaller rates for individuals with SPHC – in both residency groups. Some of these associations still turned out to be more pronounced for nursing home residents as compared to community dwellers. However, in most cases, the two groups do not differ anymore with regard to the associations between PC and healthcare indicators. This is in line with the abovementioned findings that the two groups get more similar when applying the alternative definition of groups. What remains, however, is the main effect of residency: across the board, nursing home residents still demonstrate more beneficial healthcare outcomes than community dwellers.

*Figure 1S. Adjusted rate of place of death: hospital*


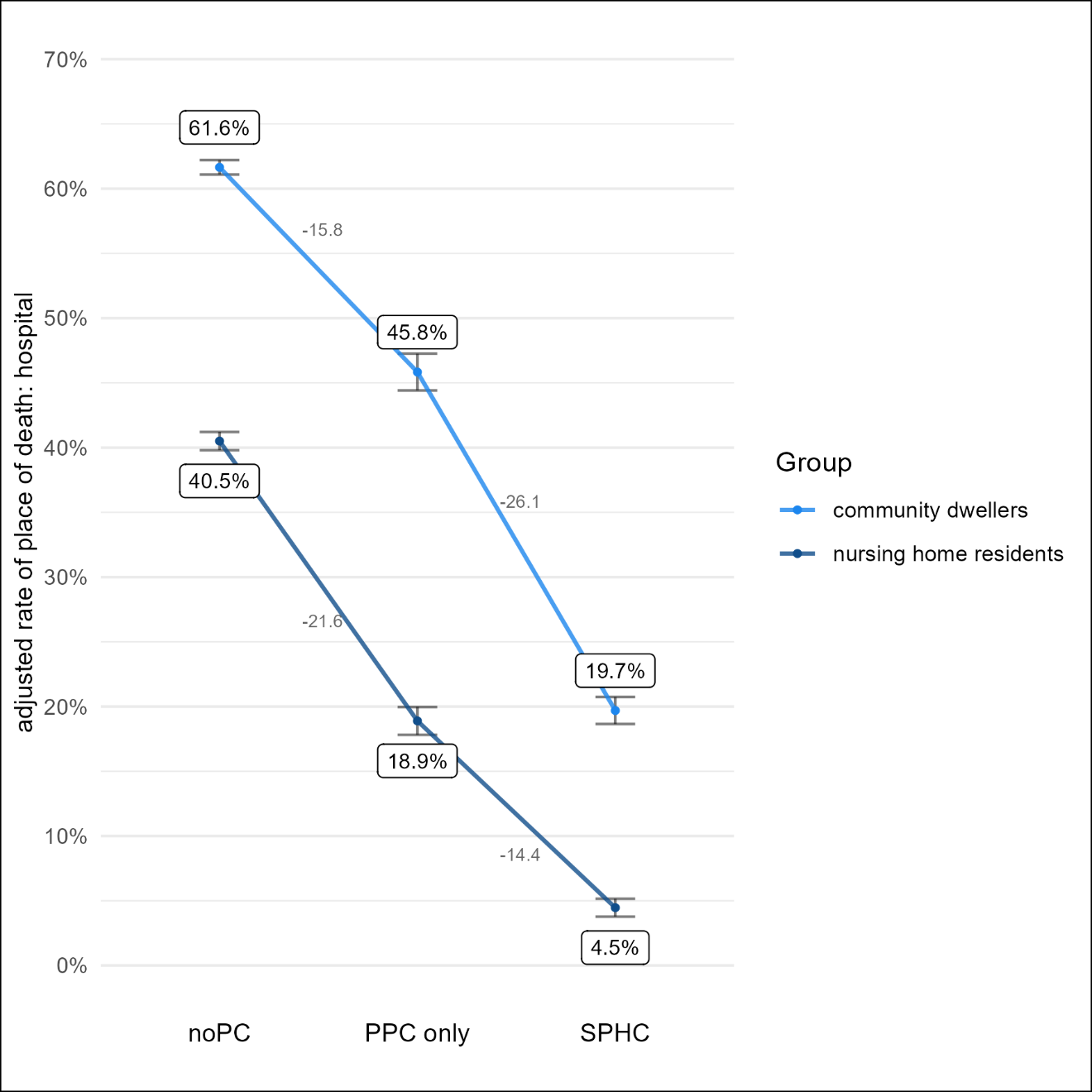


*“nursing home residents” vs. “community dwellers” according to their place of residence at the time of death only*

*Figure 2S. Adjusted rate of hospitalization*


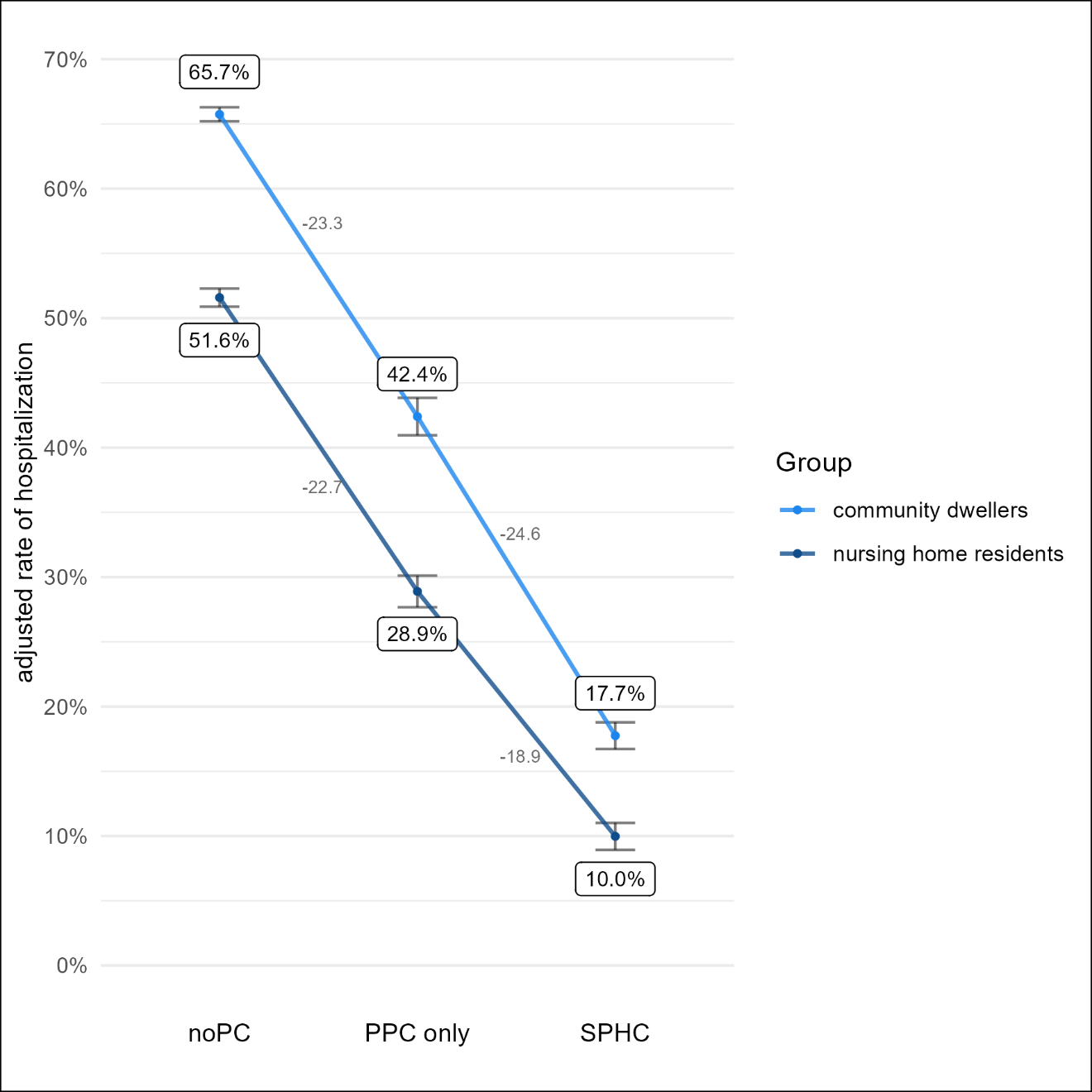


*“nursing home residents” vs. “community dwellers” according to their place of residence at the time of death only*

*Figure 3S. Adjusted rate of emergency medical services*

*
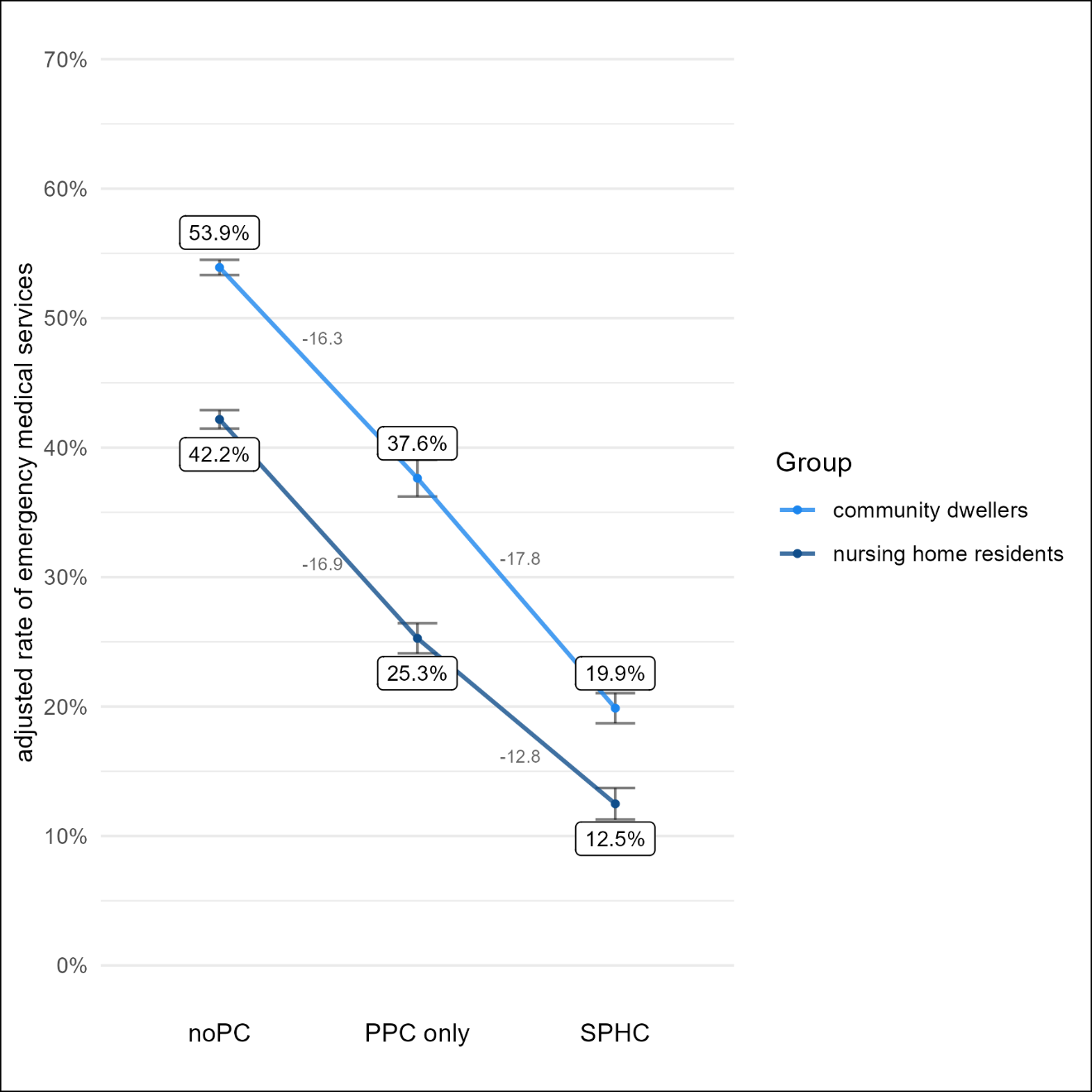
*

*“nursing home residents” vs. “community dwellers” according to their place of residence at the time of death only*

*Figure 4S. Adjusted rate of intensive care treatment*

*
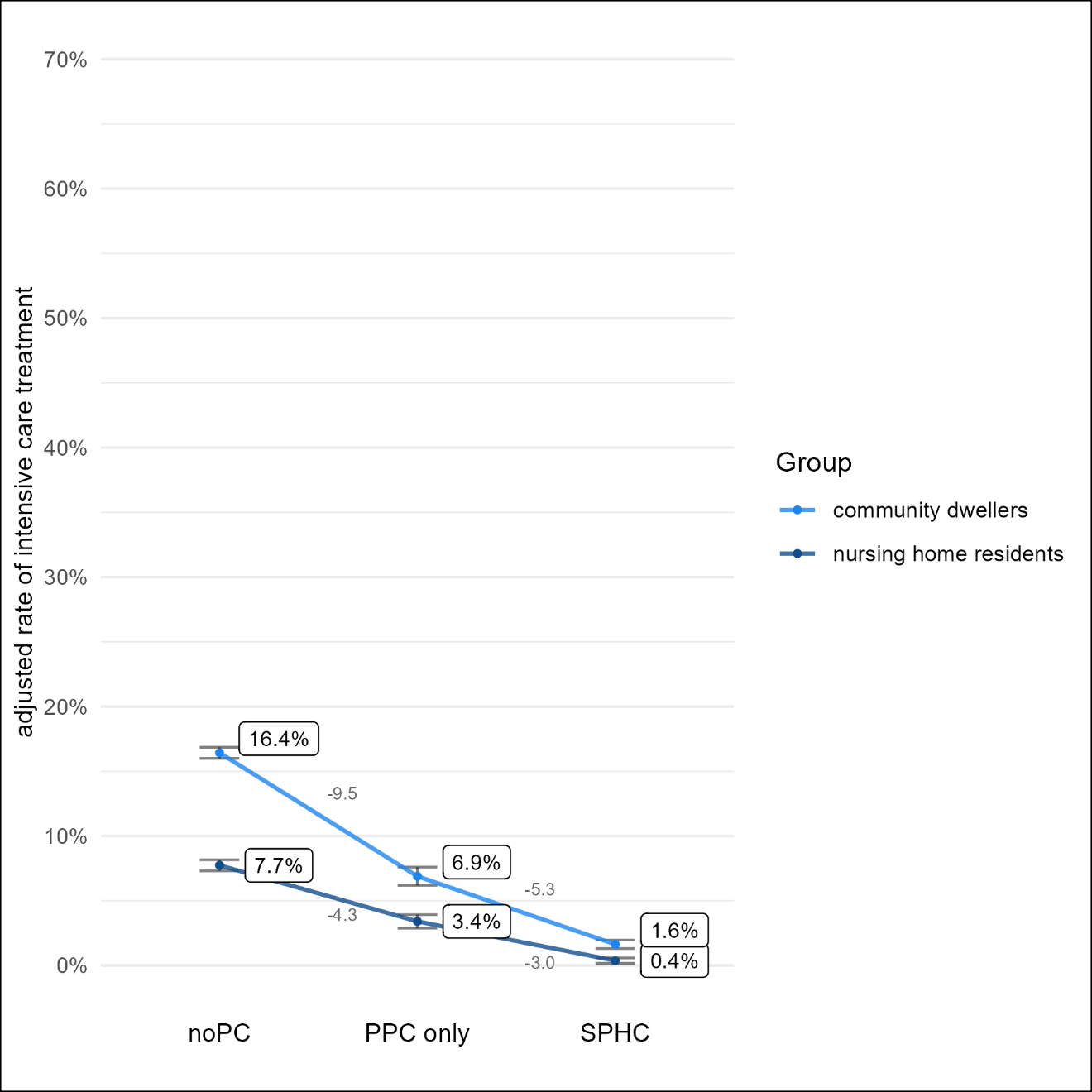
*

*“nursing home residents” vs. “community dwellers” according to their place of residence at the time of death only*

1. GKV-Spitzenverband, Kassenärztliche Bundesvereinigung (2009) Vereinbarung über die

   qualifizierte ambulante Versorgung krebskranker Patienten „Onkologie-Vereinbarung“ (Anlage

   7 zum Bundesmantelvertrag-Ärzte)
    [↑](#footnote-ref-1)
